# Supplementary material for: Content-rich biological network constructed by mining PubMed abstracts
Source: BMC Bioinformatics. 2004 Oct 8;5:147. doi: 10.1186/1471-2105-5-147 (PMC528731; doi:10.1186/1471-2105-5-147)
Supplement: Additional File 2 — The original results of the above study (non-essential files are deleted to keep the file size under the limit set by BMC bioinformatics). [file 1471-2105-5-147-S2.bz2 › chilibotAdditionalFile2/dip05/481ID8702798E10571/html/IL8RB_CXCL5.html]

 


 **IL8RB** and **CXCL5** 
  
Found 16 abstracts in PubMed, retrieved 05.  
 

 What does Google say? 
 PDF only 
| .edu only 

---

**Interactive relationship** (e.g. stimulation, inhibition, etc)

**Non-interactive relationship** (e.g. studied together, co-existance, homology, etc.)

- Thus in COPD, in addition to CXCL8 and CXCR1,  **CXCL5**  and CXCR2  [ **IL8RB** ]  appear to play important roles in the airway neutrophilia characteristic of severe exacerbations.  Ref: 12857718 Am J Respir Crit Care MedAm J Respir Crit Care Med,
- By comparison with the NSC and S COPD groups, neutrophilia and gene expression for epithelial derived neutrophil attractant 78  **CXCL5** , interleukin 8 CXCL8, CXCR1 and CXCR2  [ **IL8RB** ]  were each up regulated in the E COPD group p&lt.  Ref: 12857718 Am J Respir Crit Care MedAm J Respir Crit Care Med,
- CONCLUSION These findings suggest that, through expression of  **CXCL5** , eosinophils can recruit and activate CXC receptor 2 CXCR2  [ **IL8RB** ]  bearing cells such as neutrophils at sites of inflammation.  Ref: 12680872 Clin Exp Allergy, 2003
